# Exploring factors influencing visual disability in the elderly population of China: A nested case-control investigation

**DOI:** 10.7189/jogh.13.04142

**Published:** 2023-11-15

**Authors:** Yitian Gao, Jing Liu, Wanqiong Zhou, Jing Tian, Qiuyi Wang, Lanshu Zhou

**Affiliations:** Second Military Medical University, Shanghai, China

## Abstract

**Background:**

Factors influencing visual disability among the elderly in China remain largely unclear. We sought to determine the prevalence and identify risk factors for visual disability among older adults in China.

**Methods:**

We employed a nested case-control study design, utilising data from the China Health and Retirement Longitudinal Study (CHARLS) collected between 2011 and 2018. Cases and controls were matched by a ratio of 1:3 by age and sex. Conditional logistic regression identified factors associated with visual disability.

**Results:**

Prior to data matching, the cohort comprised 4729 complete samples, with 785 (16.6%) newly diagnosed cases of visual disability during the follow-up period. Following matching, 3132 subjects remained, with 783 in the case group and 2349 in the control group. Factors associated with the occurrence of visual disability in the elderly included per capita family income (odds ratio (OR) = 0.98; 95% confidence interval (CI) = 0.97-0.99), adequate sleep (OR = 0.75; 95% CI = 0.63-0.90), cognitive function (OR = 0.98; 95% CI = 0.96-0.99), heart disease (OR = 1.51; 95% CI = 1.20-1.89), kidney disease (OR = 1.45; 95% CI = 1.05-1.98), depression (OR = 1.04; 95% CI = 1.03-1.06), history of falls (OR = 1.34; 95% CI = 1.09-1.65), and cataracts (OR = 2.71; 95% CI = 1.81-4.07).

**Conclusions:**

Visual disability among the elderly in China remains a major concern. Per capita family income, adequate sleep, and cognitive function are protective factors, while heart disease, kidney disease, depression, history of falls, and cataracts are risk factors. Future efforts in preventing and treating visual disability in the elderly should target these high-risk factors and provide early interventions to this population.

With the global population aging and rising average life expectancy, the disease burden has shifted toward non-communicable diseases and disabilities [1,2]. It is now understood that older adults are particularly vulnerable to various types of disabilities [3]. Visual disability has been strongly associated with aging [4]. According to the latest data released by the World Health Organization (WHO), over 2.2 billion people worldwide have blindness or visual disability, with the majority being elderly individuals [5]. In China, approximately four cases of visual disability occur every minute, with around 77% of individuals with low vision being over 65 [6]. Visual disability has emerged as the third most common chronic condition leading to care dependency among the elderly. It diminishes their ability to perform daily living and increases the risk of other health issues, such as depression and fractures, resulting in a substantial burden on families and society [7]. Consequently, visual disability has become a major public health concern in China and abroad.

Visual disability refers to low vision or reduced visual field in both eyes, which cannot be restored or temporarily improved through various treatment methods, significantly impacting daily activities and social engagement [8]. Vision is vital as a sensory system, with approximately 80% of human information acquired through vision [9]. Maintaining good visual acuity is crucial for preserving daily functioning. While visual function decline is inevitable with age, the rate of vision loss varies among individuals and is influenced by a combination of individual and contextual factors [10]. The WHO estimates that 80% of visual disabilities can be prevented or treated, and the demand for eye care services is escalating due to the global aging population [11]. Therefore, it is imperative to understand the prevalence of visual disability in older adults, identify its influencing factors, and provide early interventions to reduce preventable and treatable visual disabilities, promoting eye health and healthy aging.

Visual disability is a complex geriatric health issue whose influencing factors are multidimensional and should be systematically explored. However, existing studies on visual disability in the elderly have primarily sought to explore the correlation between visual disability and single factors [12,13] or focus on the impact of diseases [14], while the factors influencing visual disability in this population have been understudied. By investigating the risk factors associated with visual disability in older adults, an early intervention targeting key factors may help maintain independence, reduce disability incidence, and decrease care dependency among older adults [15]. Therefore, our study was conducted in a large, population-based sample of older adults, aiming to identify key variables influencing visual disability through a nested case-control design and provide feasible recommendations. We hypothesise that visual disability in the elderly may be affected by various factors encompassing individual, familial, and societal aspects.

## METHODS

### Study design and data sources

We followed a nested case-control study design. Our research is a longitudinal study based on data from the China Health and Retirement Longitudinal Study (CHARLS), a national baseline survey covering 150 county-level units, 450 village-level units, and approximately 17 000 individuals in 10 000 households to collect high-quality micro-data representing middle-aged and elderly individuals and families aged 45 and above in China aiming to analyze population aging in China and promote interdisciplinary research on aging. Participants underwent face-to-face interviews conducted at their homes using computer-assisted personal interviews (CAPI) technology. The interviews gathered data on socio-demographic and lifestyle factors and health-related information. The CHARLS data was initially collected at baseline in 2011, while follow-up surveys have been conducted in 2013, 2015 and 2018. The data set includes separate weighted variables to ensure the survey sample is representative of the Chinese population. Detailed information about this database has been reported in previous studies [16].

For this study, we utilised the baseline data from 2011 and the follow-up data from 2013, 2015 and 2018 to establish an observational cohort. Before beginning our research, we obtained access to the CHARLS data from the official website (http://charls.pku.edu.cn/).

### Study participants

In accordance with the study objectives, healthy older adults without visual disability at the time of the baseline survey in 2011 were selected as study participants to establish an initial observation cohort. The following inclusion criteria were set: a) individuals aged 60 years and over, b) individuals self-reported no visual disability, meaning they answered “no” to the question “ Do you have a visual disability?” in the CHARLS survey. Exclusion criteria included samples with missing key variables in the baseline.

Specifically, 6966 elderly individuals who reported no visual disability at baseline were included. Additionally, 1603 individuals with incomplete baseline data were excluded, resulting in an initial observation cohort of 5363. The definition of elderly in this study was in accordance with the law of the People’s Republic of China on the Protection of the Rights and Interests of the Elderly, which classifies citizens over 60 years old as elderly [17]. After excluding individuals who were lost to follow-up in the surveys conducted in 2013, 2015 and 2018 (n = 634), a total of 4729 complete samples were obtained.

**Figure 1:**
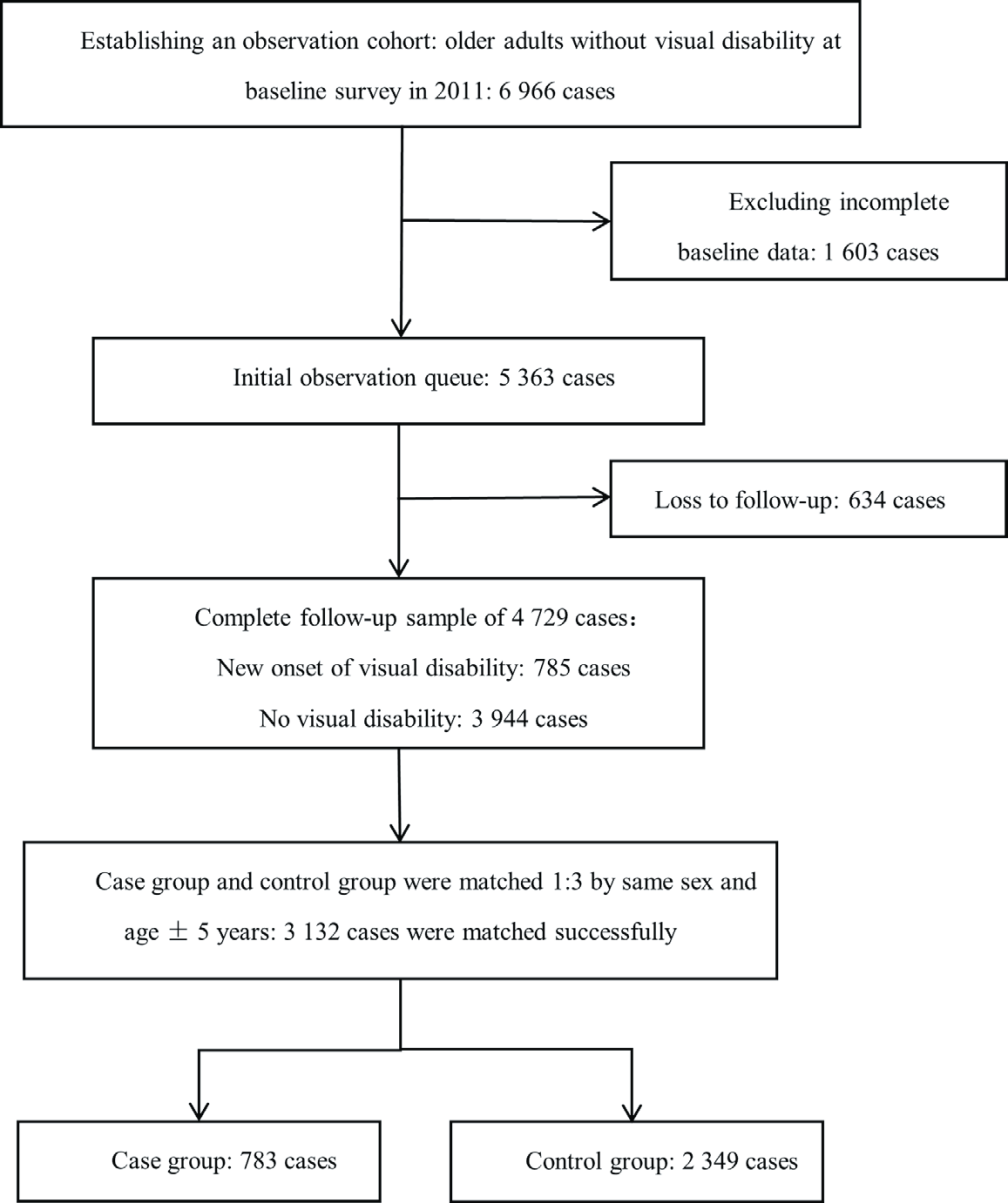
Study flow diagram.

For the case-control analysis, individuals who developed a visual disability during the three follow-up visits from 2013 to 2018 were classified as the case group, while those who did not have a visual disability before the end of the follow-up visit in 2018 were classified as the control group. Cases and controls were matched at a 1:3 ratio based on sex and age within a five-year range (**Figure 1**).

### Variables

Based on previous epidemiological data or experimental data on mechanisms of incidence of visual disability, potential factors influencing visual disability included individual, family, and social factors [18,19]. We therefore identified the relevant factors from the CHARLS database as variables for this study: the individual level (e.g. gender, age, marriage), the family level (e.g. social events, number of children, residence), and the social level (e.g. roads, supporting organisations, health service centers (**Table 1**).

**Table 1:** Assignment of variables

Individual level
Sex
*Female = 0*
*Male = 1*
Age in years
*60-64 = 1*
*65-69 = 2*
*70-74 = 3*
*75-79 = 4*
*80 and above = 5*
Marriage
*No spouse = 0*
*With spouse = 1*
Education
*Illiterate = 1*
*Elementary school = 2*
*Junior high school = 3*
*High school and above = 4*
Account
*Non-agriculture = 0*
*Agriculture = 1*
Medical insurance
*No insurance = 1*
*Urban and rural residents = 2*
*Urban employees = 3*
*Publicly funded medical insurance = 4*
*Commercial insurance = 5*
Body mass index
*Low = 1*
*Normal = 2*
*Overweight = 3*
Disability (hearing, physical, intellectual, speech)*
*No = 0*
*Yes = 1*
Comorbidities (hypertension, dyslipidaemia, diabetes mellitus, malignant tumour, chronic lung disease, liver disease, heart disease, stroke, kidney disease, digestive system disease, emotional, psychiatric disorders, memory-related diseases, arthritis, rheumatism, asthma)*
*No = 0*
*Yes = 1*
Falls
*No = 0*
*Yes = 1*
Hip fracture
*No = 0*
*Yes = 1*
Cataracts
*No = 0*
*Yes = 1*
Glaucoma
*No = 0*
*Yes = 1*
Accidental injuries
*No = 0*
*Yes = 1*
Smoking
*Never = 1*
*Quit = 2*
*Still smoking = 3*
Drinking
*No = 0*
*Yes = 1*
Napping habits
*No = 0*
*Yes = 1*
Adequate sleep (defined as more than six hours of sleep)
*No = 0*
*Yes = 1*
**Family level**
Social events
*None = 1*
*One kind = 2*
*Two kinds or more = 3*
Number of children
*None = 1*
*One child = 2*
*Two children and more = 3*
Residence
*Living with children = 1*
*Living near children = 2*
*Living away from children = 3*
Area of residence
*Rural = 1*
*Urban = 2*
Barrier-free access
*No = 0*
*Yes = 1*
Indoor toilet available
*No = 0*
*Yes = 1*
With bathing facilities
*No = 0*
*Yes = 1*
**Social level**
Mainly dirt roads
*No = 0*
*Yes = 1*
Available recreational and fitness activities
*No = 0*
*Yes = 1*
Have organisations that assist the elderly
*No = 0*
*Yes = 1*
*Have health service centres*
*No = 0*
*Yes = 1*

*Values assigned separately.

### Data analysis

We used software SPSS, version 26.0 (SPSS Foundation for IBM, New York, USA) to perform all statistical analyses. Following normality and homogeneity of variance tests, none of the data were normally distributed or met the criteria for homogeneity of variance testing. Therefore, the following statistical methods were employed for data analysis: a) descriptive analysis (median and interquartile range were used to express measurement data, while frequency and composition ratio were used to describe counting data), b) univariate analysis (Mann-Whitney U and Kruskal-Wallis H tests were used to compare classified variables), c) correlation analysis (Spearman correlation was used for the correlation analysis, d) regression analysis (the conditional logistic regression method was used to estimate the risk of visual disability in different elderly populations, and the odds ratio (OR) and confidence interval (CI) were calculated.

## RESULTS

### Characteristics of samples

Before matching the data, the cohort consisted of 4729 completed samples, 785 (16.6%) of which were newly diagnosed cases diagnosed with visual disability during the follow-up period. After matching, 3132 subjects were included, with 783 in the case group and 2349 in the control group. The case group exhibited female predominance (n = 460, 58.8%), with an age range of 60 to 91 years.

### Descriptions and correlations between visual disability and the other studied variables

As shown in Table S1 in the **Online Supplementary Document**, 26 variables with significant differences between the two groups included: a) individual level (account, education, medical insurance, comorbidities (chronic lung disease, liver disease, heart disease, kidney disease, digestive system disease, rheumatism), intellectual disability, cognitive function, depression, physical mobility, body mass index (BMI), history of disability-related illness (falls, cataracts, glaucoma), adequate sleep, and napping habit); b) family level (per capita family income, area of residence, and bathing facilities) c) social level (community environment (mainly dirt roads, recreational and fitness activities, organisations that assist the elderly, health service centers)).

### Indicators of influencing factors of visual disability among the elderly

A conditional logistic regression analysis of the Cox risk regression model was conducted using visual disability as the dependent variable (coded as one for the case group and zero for the control group). The 26 variables with significant differences between the two groups mentioned above were used as independent variables. The conditional logistic regression analysis using stepwise regression with an inclusion criterion of 0.05 and an exclusion criterion of 0.10 yielded significant results (**Table 2**).

**Table 2:** Results of conditional logistic regression analysis of factors influencing visual disability in the elderly

Variables	OR (95% CI)	*P*-value
Per capita family income (US$1000)	0.98 (0.97-0.99)	0.001
Adequate sleep	0.75 (0.63-0.90)	0.001
Cognitive function	0.98 (0.96-0.99)	0.014
Heart disease	1.51 (1.20-1.89)	<0.001
Kidney disease	1.45 (1.05-1.98)	0.022
Depression	1.04 (1.03-1.06)	<0.001
Falls	1.34 (1.09-1.65)	0.006
Cataracts	2.71 (1.81-4.07)	<0.001

OR – odds ratio, CI – confidence interval

The variables that showed a statistically significant impact on the occurrence of visual disability in the elderly were per capita family income (OR = 0.98; 95% CI = 0.97-0.99), adequate sleep (OR = 0.75; 95% CI = 0.63-0.90), cognitive function (OR = 0.98; 95% CI = 0.96-0.99), heart disease (OR = 1.51; 95% CI = 1.20-1.89), kidney disease (OR = 1.45; 95% CI = 1.05-1.98), depression (OR = 1.04; 95% CI = 1.03-1.06), history of falls (OR = 1.34; 95% CI = 1.09-1.65), and cataracts (OR = 2.71; 95% CI = 1.81-4.07).

Among these variables, per capita family income, adequate sleep, and cognitive function were protective factors. Specifically, for every US$1000 increase in family income, the risk of visual disability in the elderly decreased by 2%. Elderly individuals who reported sufficient sleep at night had a 25% lower risk of visual disability than those with insufficient sleep. Moreover, elderly individuals with good cognitive function exhibited a 2% reduced risk of visual disability compared to those with poor cognition. On the other hand, the risk of visual disability was found to be higher for elderly individuals with heart disease (51% higher risk), kidney disease (45% higher risk), depression (4% higher risk), and a history of falls (34% higher risk). Additionally, elderly individuals with cataracts had nearly twice the risk of visual disability compared to those without cataracts.

In response to the issue of missing data deletion in this study, sensitivity analysis was implied to determine the stability of the results. After using multiple imputations to process incomplete data, conditional logistic regression analysis of the Cox risk regression model was performed on the imputed data. There were no substantial changes in the results, indicating that the results of this study are stable (Table S2 in the **Online Supplementary Document**).

## DISCUSSION

We aimed to examine the prevalence and identify the risk factors of visual disability among the elderly in China. Our findings revealed a prevalence rate of 16.6% for visual disability among the elderly population in China, higher than reported in a review by Zou et al. [20], which reported prevalence rates of 10.9% for moderate to severe visual impairment and 2.2% for blindness among the elderly in China.

The heterogeneity in prevalence rates may be attributed to the differences in the study populations. Zou et al. included individuals aged 50 and older, while this study focused on individuals aged 60 years or above. It is worth noting that visual disability shows a strong correlation with age, indicating that as individuals grow older, the likelihood of experiencing visual disability increases [21]. The results of this study highlighted the relatively high prevalence of visual disability among the elderly in China. Accordingly, conducting further analysis of key variables that influence the development of visual disability in older adults and implementing early preventive interventions can be an effective approach to reducing visual disability among this population.

This study found that per capita family income, adequate sleep, and cognitive function were protective factors against visual disability in older adults, whereas heart disease, kidney disease, depression, history of falls, and cataracts were risk factors. Consistently, a study by Wong et al. [22] on Asian populations noted that low income explained 58.1% of best-corrected visual acuity loss and that older adults with high per capita family income were less likely to experience visual disability. On the one hand, low income implies poorer economic resources and thus poorer accessibility and effectiveness of eye care services; on the other hand, due to financial constraints, older adults in low-income households tend to have lower medical accessibility, resulting in greater vulnerability to vision problems [23]. To address this issue, the local authorities need to improve current health care policies and access to medical subsidies and other related benefits for economically disadvantaged older adults [24]. By reducing financial burdens and providing necessary support, these policies can assist in addressing the difficulties faced by the elderly population and improve their overall well-being.

The present study highlighted the association between sleeping over six hours and a lower likelihood of developing visual disability in older adults. It is widely acknowledged that sleep plays a vital role in the physiological recovery of the body, and insufficient sleep duration may impact endocrine metabolism, consequently increasing the risk of various diseases [25]. Huang et al. study [26] demonstrated that sleep is a complex behavior influenced by various factors, such as environment, personal habits, anxiety levels, etc. Therefore, to improve sleep quality in older adults, it is essential to consider individual characteristics and the effects of diseases, mental health, and other related factors on sleep. Providing a comfortable sleep environment and fostering good sleep habits are important, but a comprehensive approach that addresses the multifaceted nature of sleep is necessary to optimise sleep quality in older adults.

Our research findings indicated that elderly individuals with heart or kidney diseases were at a higher risk of experiencing visual disability. Heart disease and kidney disease were prevalent chronic conditions among older adults, consistent with the findings of Fong et al. study [27] on elderly individuals in the USA, which showed that older adults with major non-communicable diseases were more prone to developing disability issues at an earlier stage. From a physiological perspective, Wong et al. [22] found that chronic kidney disease was closely related to retinopathy, which may lead to visual disability. Additionally, heart disease, including coronary heart disease and heart failure, can lead to vascular damage and increased blood clotting, elevating the risk of retinal vascular occlusion and subsequent visual disability [28]. These results indicate that strengthened visual function assessment and health education for older adults with cardiovascular and renal conditions is imperative, as they represent high-risk groups for age-related visual disability.

It is now understood that depression is a common emotional disorder that significantly impacts the well-being of elderly individuals, increasing the risk of disability and reducing overall health recovery and quality of life [29,30]. Our study demonstrated a close association between depression in the elderly and the occurrence of visual disability. Szakáts study [31] revealed that depression could increase somatosensory sensitivity and even corneal nerve hypersensitivity, thereby affecting visual function among the elderly. Therefore, it is recommended to enhance the evaluation mechanism and support system for the mental health of older adults [32]. Wide-ranging mental health education activities should be implemented to encourage active participation in social activities and promote mental well-being among the elderly. These measures can contribute to better supporting healthy aging and improving the overall quality of life for older individuals [33].

The research findings emphasised that cataracts posed a significant risk for visual disability among elderly individuals. Despite being highly treatable, cataracts remained a leading cause of visual disability and blindness globally [34]. Both domestic and international studies [35,36] have consistently shown that proactive cataract prevention and early surgical intervention are the most effective approaches for managing cataracts. However, a significant proportion of elderly people lack an understanding of cataract-related knowledge, leading to insufficient attention and rejection of surgical treatment, ultimately leading to severe visual impairment [37]. With a view to improving this problem, it is crucial for relevant health departments and community nursing staff to enhance patient education regarding the causes of cataracts and the benefits of surgical treatment [38]. By providing comprehensive and accurate information on the feasibility and safety of cataract treatment, patients can develop a better understanding and awareness, thereby avoiding avoidable visual impairment and making informed decisions regarding their eye health.

In addition, this study found that elderly people with a history of falls were more likely to develop visual disability. As people age, their physical functions deteriorate, and the increased risk of falls becomes inevitable [39]. Many studies have reported the correlation between visual function and falls, and visual disability is a high-risk factor for increased falls. However, further exploration is needed to determine whether falls are a risk factor for visual disability.

Interestingly, the results of this study revealed that good cognitive function was also a protective factor for visual disability, suggesting that elderly people with good cognitive function have a lower risk of developing visual disability [40,41]. However, the underlying mechanism for this relationship remained unclear and was not fully elucidated in this study. It is worth noting that existing research has primarily focused on investigating the impact of visual function on cognitive function, with limited studies exploring the reverse relationship of cognition affecting vision [42]. This indicates a research gap in understanding the specific mechanisms involved. Further research efforts in this direction would contribute to a more comprehensive understanding of the interplay between cognition and vision, shedding light on the underlying mechanisms and potential pathways.

### Limitations

This study has some limitations. First, visual disability in the elderly was assessed through self-report, which introduced the possibility of bias. Second, the underlying mechanisms by which variables like falls and cognitive function influenced visual disability in the elderly remained unclear. Despite their significant associations, the specific pathways and causal relationships were not fully understood. Further research is needed to explore the role relationships among these variables. Third, the study’s findings were limited to a specific population and restricted to a single race and country. Therefore, caution should be exercised in generalising the results to other populations or diverse demographic backgrounds. Lastly, in order to fully utilise these data, we did not analyze variables such as screening time that may affect the results, which could have biased the results to a certain extent.

## CONCLUSIONS

Overall, visual disability remained a significant issue among elderly individuals in China, with a notable prevalence. Factors such as per capita family income, adequate sleep, and cognitive function protect against visual disability in the elderly. Conversely, risk factors include heart disease, kidney disease, depression, history of falls, and cataracts. Future efforts in preventing and treating visual disability in the elderly should focus on these high-risk factors and provide early interventions to protect this population.

## Additional material


Online Supplementary Document
