# Supplementary material for: Exploring factors influencing visual disability in the elderly population of China: A nested case-control investigation
Source: J Glob Health. 2023 Nov 15;13:04142. doi: 10.7189/jogh.13.04142 (PMC10644848; doi:10.7189/jogh.13.04142)
Supplement: Online Supplementary Document [file jogh-13-04142-s001.pdf]

Table S1. Descriptive statistics and univariate analysis of baseline data（*n*=3132）

Variables		Case group（n=783）		Control group（n=2349）		Statistics	*P*
		N	Ratio (%)	N	Ratio (%)		
Age (year)	60-65	252	32.2	766	32.6	0.232(H)	0.630
	65-70	220	28.1	670	28.5		
	70-75	156	19.9	479	20.4		
	75-80	102	13	276	11.7		
	80 and above	53	6.8	158	6.7		
Sex	Female	460	58.7	1355	57.7	-0.522(Z)	0.601
	Male	323	41.3	994	42.3		
Account	Agriculture	659	84.2	1824	77.7	-3.894(Z)	**<0.001 **
	Non-agriculture	124	15.8	525	22.3		
Marriage	No spouse	168	21.5	523	22.3	-0.473(Z)	0.636
	With spouse	615	78.5	1826	77.7		
Education	Illiterate	350	44.7	891	37.9	19.279(H)	**<0.001 **
	Elementary School	342	43.7	1027	43.7		
	Junior High School	57	7.3	278	11.8		
	High School and above	34	4.3	153	6.5		
Medical Insurance	No insurance	42	5.4	150	6.4	5.601(H)	**0.018***
	Urban and rural residents	663	84.7	1842	78.4		
	Urban employees	51	6.5	255	10.9		
	Publicly funded medical insurance	15	1.9	62	2.6		

	Commercial insurance	12	1.5	40	1.7		
Per capita family income (thousand dollars)		2.46（0.72，6.81）		3.27（0.80，9.00)		-.065(r)	**<0.001** **
Comorbidities	Hypertension	254	32.4	726	30.9	-0.801(Z)	0.423
	Dyslipidemia	82	10.5	235	10	-0.376(Z)	0.707
	Diabetes mellitus	65	8.3	163	6.9	-1.27(Z)	0.204
	Malignant tumor	9	1.1	18	0.8	-1.004(Z)	0.315
	Chronic lung disease	126	16.1	288	12.3	-2.741(Z)	**0.006****
	Liver disease	45	5.7	77	3.3	-3.092(Z)	**0.002****
	Heart disease	150	19.2	315	13.4	-3.916(Z)	**<0.001** **
	Stroke	28	3.6	70	3	-0.829(Z)	0.407
	Kidney disease	72	9.2	124	5.3	-3.918(Z)	**<0.001** **
	Digestive system disease	212	27.1	475	20.2	-4.013(Z)	**<0.001** **
	Emotional/ psychiatric disorders	8	1	29	1.2	-0.477(Z)	0.633
	Memory-related diseases	18	2.3	39	1.7	-1.157(Z)	0.247
	Rheumatism	372	47.5	863	36.7	-5.34(Z)	**<0.001** **
	Asthma	47	6	107	4.6	-1.622(Z)	0.105
Disability	Hearing disability	92	11.7	237	10.1	-1.312(Z)	0.190
	Physical	30	3.8	86	3.7	-0.218(Z)	0.827

	disability						
	Intellectual disability	24	3.1	38	1.6	-2.518(Z)	**0.012***
	Speech disability	1	0.1	6	0.3	-0.655(Z)	0.512
Cognitive function		9.00（5.00，13.00）		11.00（6.00，15.00）		-.086(r)	**<0.001 ****
Depression level		10.00（6.00，16.00）		7.00（4.00，12.00）		.166(r)	**<0.001 ****
Physical mobility		6.00（5.00，8.00）		7.00（5.00，8.00）		-.060(r)	**<0.001 ****
BMI	Low	98	12.5	234	10	6.723(H)	**0.010***
	Normal	416	53.1	1204	51.3		
	Overweight	269	34.4	911	38.7		
History of disability-related illness	Accidental Injuries	65	8.3	213	9.1	-0.653(Z)	0.514
	Falls	197	25.2	424	18.1	-4.32(Z)	**<0.001 ****
	Hip fracture	12	1.5	42	1.8	-0.475(Z)	0.634
	Cataracts	49	6.3	63	2.7	-4.666(Z)	**<0.001 ****
	Glaucoma	15	1.9	19	0.8	-2.588(Z)	**0.010***
Adequate sleep	Yes	352	45	776	33	-6.016(Z)	**<0.001 ****
	No	431	55	1573	67		
Napping habit	Yes	391	49.9	1069	45.5	-2.15(Z)	**0.032***
	No	392	50.1	1280	54.5		
Social events	None	404	51.6	1196	50.9	0.362(H)	0.547
	one kind	283	36.1	831	35.4		
	two kinds and	96	12.3	322	13.7		

	more						
Smoking	Never	498	63.6	1459	62.1	0.24(H)	0.624
	Quit	68	8.7	244	10.4		
	Still smoking	217	27.7	646	27.5		
Drinking	Yes	530	67.7	1566	66.7	-0.818(Z)	0.413
	No	253	32.3	783	33.3		
Number of children	None	11	1.4	52	2.2	0.04(H)	0.841
	One	64	8.2	177	7.5		
	Two and more	708	90.4	2120	90.3		
Seeing children often	Yes	352	45	981	41.8	-1.565(Z)	0.118
	No	431	55	1368	58.2		
Residence	Living with children	336	42.9	1068	45.5	2.402(H)	0.121
	Living near children	374	47.8	1103	47		
	Living away from children	73	9.3	178	7.6		
Area of residence	Rural	672	85.8	1834	78.1	-4.694(Z)	**<0.001 ** **
	Urban	111	14.2	515	21.9		
Home environment	Barrier-free access	201	25.7	584	24.9	-0.452(Z)	0.651
	Indoor toilet available	563	71.9	1665	70.9	-0.546(Z)	0.585
	With bathing facilities	191	24.4	726	30.9	-3.468(Z)	**0.001 ** **
Community environment	Mainly dirt roads	210	26.8	496	21.1	-3.308(Z)	**0.001 ** **
	Available recreational and	489	62.5	1583	67.4	-2.529(Z)	**0.011***

	fitness activities						
	Have organizations that assist the elderly	214	27.3	743	31.6	-2.262(Z)	**0.024***
	Have health service centers	617	78.8	1759	74.9	-2.218(Z)	**0.027***

N – Number; r – Spearman Correlation Analysis; Z – Mann-Whitney U-test;

H – Kruskal-Wallis H-test.

* Correlation is significant at 0.05 level; **Correlation is significant at 0.01 level.

Sensitivity analysis of missing data

In this study, 6966 older adults without visual disability were screened from the database at the beginning, of which 634 cases were lost in follow-up and had missing data for all variables, which made interpolation impossible, and were therefore directly censored. Another 1,603 older adults were missing both general information and variable data to varying degrees, of which 790 older adults had more than 90% missing data and were not suitable for interpolation, so only the remaining 813 older adults were interpolated using multiple interpolation in this study.

After using multiple imputation to process incomplete data, conditional logistic regression analysis of the Cox risk regression model was performed on the imputed data. There were no substantial changes in the results, indicating that the results of this study are stable (see **Table S2**).

Table S2. Results of Multivariant analysis of Missing Data

**Variables**	***P***	**OR**	**95% CI**	
			**lower**	**upper**
Per capita family income	<0.001	0.98	0.97	0.99
Sleeping over 6 hours	<0.001	0.76	0.66	0.87
Cognitive function	0.001	0.98	0.97	0.99
Heart disease	0.004	1.29	1.08	1.53
Kidney disease	0.039	1.28	1.01	1.63
Depression	<0.001	1.03	1.02	1.04
Falls	0.009	1.23	1.06	1.44
Cataracts	<0.001	2.16	1.64	2.84

OR – Odds Ratio; CI – Confidence Interval.
